# Test–Retest Reliability of TRF-Derived Measures of Cortical Tracking of the Speech Envelope

**DOI:** 10.1523/ENEURO.0068-25.2025

**Published:** 2025-08-13

**Authors:** Heather R. Dial, G. Nike Gnanateja

**Affiliations:** ^1^Department of Communication Sciences and Disorders, University of Houston, Houston, Texas 77204; ^2^Department of Communication Sciences and Disorders, University of Wisconsin-Madison, Madison, Wisconsin 53706

**Keywords:** broadband envelope, cortical tracking, envelope tracking, multiband envelope, temporal response function, test–retest reliability

## Abstract

Cortical tracking of the speech envelope is an emerging, noninvasive measure of neurophysiological processing of speech that is being widely adopted. It demonstrates good ecological validity, as it allows researchers to study human processing of continuous, naturalistic speech containing dynamic spectrotemporal variations and rich linguistic content. While measures of cortical tracking have strong clinical and research applications, there is a lack of research documenting the reliability of these measures, including how they are affected by the stimulus and how the stimulus is represented, as well as electroencephalography (EEG) acquisition and analysis parameters. In this study, we measured the test–retest reliability of cortical tracking of the speech envelope across different stimuli (an audiobook vs a podcast), stimulus features (broadband envelope and its derivative, multiband envelope and its derivative), reference electrodes (average mastoid vs common average reference), and EEG frequency bands (delta, theta, full) in 26 neurotypical adults (20 female) to assess the potential for cortical tracking to serve as a reliable measure of speech processing. We also examined the amount of data required to achieve stable reliability estimates. We observed moderate to good test–retest reliability for most parameters with as little as 390 s of data, supporting the utility of cortical tracking of the speech envelope as a reliable tool for assessing speech processing. The findings of this study will allow researchers to more effectively design and implement studies on cortical tracking in neurotypical adults and adults with language disorders.

## Significance Statement

This study highlights the importance of cortical tracking of the speech envelope as a reliable, noninvasive measure of how the brain processes speech. By demonstrating moderate to good test–retest reliability across various stimuli, features, reference electrodes, and electroencephalography frequency bands with as little as 390 s of data, the research supports the use of cortical tracking in both clinical and research settings. These findings will help researchers design better studies on speech processing in both neurotypical adults and those with language disorders, ultimately advancing our understanding of neural processing of speech and language.

## Introduction

Auditory comprehension deficits exist across a range of communication disorders (Mandal et al., 2016; Di Liberto et al., 2018; Lwi et al., 2021). Using controlled tasks and stimuli (e.g., minimal pairs discrimination, single picture–word verification) helps to identify the level(s) at which deficits exist, but such tasks may lack ecological validity as we do not typically communicate using syllables, words, or even sentences in isolation. Moreover, due to task confounds, several tasks are necessary for the precise identification of deficits (Dial and Martin, 2017), potentially requiring hours of testing, which is not clinically feasible and leads to fatigue and frustration. This poses a critical barrier in the comprehensive characterization of comprehension deficits, hindering scientific progress and speech–language intervention. With recent technological advances, however, it is possible to objectively measure neural correlates of auditory comprehension across levels of processing using a single, naturalistic task (Crosse et al., 2016, 2021; Gnanateja et al., 2022) where participants listen to a continuous narrative while electroencephalography (EEG) responses are recorded. Cortical tracking (i.e., alignment of neural oscillations with an incoming stimulus) of acoustic and linguistic features of the narrative is then examined.

Many studies on cortical tracking examined the speech envelope, which contains acoustic cues for pitch, and syllable, word, and phrase boundaries (Oganian and Chang, 2019). Aberrant tracking of the speech envelope has been observed in individuals with hearing loss (Fuglsang et al., 2020), logopenic variant primary progressive aphasia (Dial et al., 2021), and stroke-induced aphasia (Kries et al., 2023), suggesting sensitivity to changes in speech processing in clinical populations. More broadly, cortical tracking research helps to inform our understanding of the cognitive and neural bases of speech understanding (Ding and Simon, 2014; Kaufeld et al., 2020; Hamilton et al., 2021; Coopmans et al., 2022; Ten Oever et al., 2022).

Several methods have been used to assess cortical tracking of speech (Gnanateja et al., 2022), including temporal response function (TRF) modeling, which involves deriving a linear function to map acoustic and linguistic features of a stimulus to neurophysiological data. TRF-predicted EEG responses for portions of the stimulus not used in the derivation of the function are correlated with the observed EEG, with the strength of the correlation reflecting the strength of the neural representation of that feature. With widely available tools to estimate TRFs (mTRF Toolbox, Crosse et al., 2016; EELBRAIN, Brodbeck et al., 2023), TRF modeling is increasingly being used. However, the clinical and scientific potential of this approach is dependent upon its test–retest reliability.

Recent research indicates good reliability for tracking noise (Cabral-Calderin and Henry, 2022) and moderate reliability for tracking the broadband amplitude-onset envelope of podcasts and an in-house recorded story (Panela et al., 2024). However, previous research has not examined the reliability of different speech envelope models, EEG frequency bands, or EEG reference electrodes. Furthermore, previous research presented each stimulus in its entirety (7–10 min; Panela et al., 2024), which could be problematic in clinical populations as they may struggle to maintain attention. The current study sought to fill these gaps by examining test–retest reliability of cortical tracking measures across different stimuli (audiobook, podcast), stimulus features (broadband and multiband envelopes and their derivatives), reference electrodes (average mastoid, common average), and EEG frequency bands (delta, theta, combined delta–theta) using an experimental procedure that is more appropriate for a clinical population. Our research questions were as follows:

1. What is the test–retest reliability of TRF-derived measures of cortical tracking of the speech envelope in neurotypical adults in the delta and theta bands? Given that most studies have not examined the delta and theta bands separately, we also examined the “full” band (1–8 Hz) for comparison.
2. Are there differences in test–retest reliability depending on the nature of the stimulus or how it is modeled?
3. Does the choice of reference impact test–retest reliability?

To answer these questions, we examined prediction accuracy of the TRF model and the time-course of the TRF.

## Materials and Methods

### Participants

Thirty-one neurotypical adults were enrolled in the study. Two participants were unable to return for a second session, and data from two participants could not be used due to recording errors; an additional participant was excluded as it was determined that the participant did not meet the inclusion criteria due to a (previously undisclosed) developmental language disorder. Thus, data from 26 participants were analyzed in the current study (20 females; *M*_age in years_ = 31.23; SD = 14.09; range, 18.58–82.14). Participants were recruited through the University of Houston via flyers and the research management system, SONA, as well as through Research Match (researchmatch.org) and word of mouth. Participants provided written informed consent and were compensated $40 per session or given course credit. This study was approved by the Institutional Review Board at the University of Houston.

### Materials and procedure

Participants listened to 30 ∼1 min (65 s) segments of an audiobook (*Who Was Albert Einstein?*; Brallier, 2002) and 12 ∼1 min segments (65 s) of a podcast (*My Day with the Yankees*, from the Moth Radio Hour; McGough, 2015) at two time-points separated by at least 1 week (*M*_days_ = 47.54; SD = 56.18; range, 6–230). Note that two participants completed the second session 229 and 230 d after their first session, whereas all other participants completed their second session within 70 d of their initial session. Excluding these two participants, the average number of days between sessions was 32.38. Each segment started and ended with a complete sentence. In Session 1, participants were asked a multiple-choice question after each segment of the audiobook to encourage attending to the story. A subset of participants (*n* = 6) also responded to questions in Session 2. After the question was answered or, when there were no questions, after the audio ended, participants were given the opportunity to take a break of their chosen duration and told to press the spacebar on a keyboard to continue to the next trial. Audio was presented through Sennheiser HD280 Pro headphones. The sound intensity was adjusted as needed to the participants’ comfort levels, typically between 65 and 75 dB SPL for the audiobook and 55–60 dB SPL for the podcast. All participants first listened to the audiobook, followed by a break for the participant's chosen duration before listening to the podcast.

### EEG acquisition and preprocessing

While participants listened to the audiobook and podcast, EEG data were acquired using a 64-channel (extended 10–20 international system) active electrode system (BrainProducts actiCHamp Plus) with a 25,000 Hz sampling rate. The audio that participants listened to was also recorded through the EEG system; specifically, the audio was sampled at 25,000 Hz using a BrainProducts StimTrak, to adjust for trigger timing offsets and to monitor the stimulus intensity level. All electrode impedances were under 15 kΩ.

Data were preprocessed using EEGLAB 2019.0 in MATLAB 2021b. First, data were resampled to 128 Hz and then filtered from 1 to 15 Hz. A noncausal, Hamming windowed-sinc FIR filter was used for filtering (high-pass filter cutoff, 1 Hz; filter order, 846; low-pass filter cutoff, 15 Hz; filter order, 212). Channels with activity more than three standard deviations from surrounding channels were rejected and replaced via spherical spline interpolation. Artifact subspace reconstruction (ASR) was used to suppress large artifacts (Mullen et al., 2015). ASR uses adaptive spatial filtering to remove high-amplitude burst artifacts while recovering cleaner EEG data. This was performed using a sliding-window principal component analysis, which was then used to statistically interpolate high-variance signal components exceeding a threshold relative to the covariance in a calibration dataset. Approximately 60 s of clean data were manually identified and input as the calibration data for ASR. ASR-cleaned data were epoched from −5 to 70 s relative to the stimulus onset. Data were referenced to the average of the two mastoids, and independent component analysis (ICA) was performed to correct eye movement, muscle, and electrocardiographic artifacts. ICA was performed using infomax algorithm runica.m, adjusted to extract 30 components in order to meet full rank-order assumptions of ICA. Components were removed based on manual inspection of time-course, topography, and spectrum. The resulting data comprised our “average mastoid” reference condition. Given our interest in determining whether the choice of EEG reference influences test–retest reliability, an additional reference was examined, the “common average reference” (CAR). The mastoid-referenced data were rereferenced to the CAR (average across all channels), and the resulting data comprised our CAR condition. Given the proposed differences in processing in the delta and theta frequency ranges, the data for both EEG reference conditions were filtered once more during the TRF estimation into the delta band (1–4 Hz), theta band (4–8 Hz), and full band (1–8 Hz).

### TRF modeling

#### Stimulus models

*Broadband envelope and broadband envelope derivative*. The broadband envelope was generated by taking the absolute value of the Hilbert transform of the auditory stimuli from 250 to 8,000 Hz. The envelope was then raised to a power of 0.6 to mimic the compression characteristics of the inner ear (Vanthornhout et al., 2018). Given that the auditory cortex is more sensitive to acoustic edges than sustained stimulus features (Hamilton et al., 2018), we also computed a broadband envelope derivative. This was accomplished by calculating the first temporal derivative of the broadband envelope. These edges denote rapid amplitude changes and primarily indicate the onsets and offsets of acoustic events such as phonemes and syllables.

*Multiband envelope and multiband envelope derivative*. To generate the multiband envelope, we filtered the auditory stimuli using a bank of eight gammatone filters, evenly distributed on an equivalent rectangular bandwidth scale from 250 to 8,000 Hz (Slaney, 1998). The multiband envelope consisted of the absolute value of the Hilbert transform for each of these eight bands, which was then raised to a power of 0.6. The resulting eight band-specific speech envelopes were *z*-scored within each band. As with the broadband envelope, we also computed a multiband envelope derivative by taking the first temporal derivative of the eight band-specific envelopes.

#### TRF estimation

TRF estimation was conducted for three frequency bands: delta (1–4 Hz), theta (4–8 Hz), and full (1–8 Hz). This was achieved using a forward modeling approach implemented in the mTRF toolbox (mTRF_v1.4; Crosse et al., 2016). Prior to estimating the TRFs, each participant's EEG data for each channel were *z*-scored to the mean of all channels. TRFs were estimated separately for the audiobook and podcast. The TRF was estimated by minimizing the least squares distance between the EEG predicted from the time-lagged features of the speech envelopes (−100 to 1,000 ms) and the observed EEG. To prevent overfitting and smooth the TRFs, we applied ridge regularization, with the optimal ridge parameter estimated individually for each participant. Leave-one-out cross–validation was employed to further reduce overfitting (30-fold for the audiobook, 12-fold for the podcast). In this cross-validation procedure, the TRFs from *n* tracks were used to predict the EEG in the *k*th track. This was iterated to obtain the prediction accuracy for each track for each electrode. Cortical tracking was operationally defined as the mean prediction accuracy of the TRF model across tracks, where prediction accuracy was quantified as Pearson's correlation between the observed and TRF-predicted EEG. To determine chance-level prediction, the TRF was estimated for 100 permutations of mismatched stimulus and EEG for each participant. For each participant, we examined the observed prediction accuracy relative to the chance prediction accuracy for that participant, in addition to examining the median prediction accuracy across participants relative to the mean chance prediction accuracy across participants.

Lastly, to evaluate the effect of the amount of data on the intraclass correlation coefficients (ICC) for the prediction accuracies across the different stimulus features, bands, and reference strategies, we reran our TRF estimation using between 3 and 30 (audiobook) or 12 (podcast) 65-s-long segments of data, in one segment intervals. We computed the prediction accuracy at Session 1 and Session 2 for all the new TRFs and examined test–retest reliability for each new TRF.

### Experimental design and statistical analysis

Our first research question was whether the TRF-derived measures were reliable in the delta, theta, and full bands. We assessed test–retest reliability across sessions in a number of ways, including calculating ICCs on the TRF prediction accuracies for Session 1 and Session 2; calculating ICCs on the time-course of the TRF for Session 1 and Session 2; examining prediction accuracy using Bland–Altman plots, which display agreement across sessions; and running linear mixed-effect regression models with prediction accuracy as the dependent variable and session (1 vs 2) and relevant interactions (see below) as predictors. For ICC calculations, within-subject ICCs were calculated using single measurement, absolute agreement, two-way mixed–effect models (Koo and Li, 2016). Within-subject ICC calculation allows for the assessment of reliability across sessions rather than reliability across participants. ICCs were calculated separately for each electrode channel for each stimulus (story, podcast), stimulus feature (broadband envelope and its derivative, multiband envelope, and its derivative), reference condition (average mastoid, CAR), and EEG frequency band (delta, theta, full). ICCs were calculated both for prediction accuracy (Session 1 vs Session 2) and the time-course of the TRFs. For TRF time-course ICCs, the ICC was calculated for each time point across the time interval from −50 to 950 ms. For our evaluation of the effect of the amount of data on test–retest reliability, only prediction accuracy ICCs were examined. ICCs are reported for channel Cz for brevity. Results for all electrodes are available at https://osf.io/pjau8/. ICC interpretation was as follows: >0.90 indicated excellent reliability, between 0.75 and 0.90 indicated good reliability, between 0.50 and 0.75 indicated moderate reliability, and <0.50 indicated poor reliability (Koo and Li, 2016). Bland–Altman plots are presented as an additional means of displaying the agreement across sessions. In Bland–Altman plots, the *x*-axis represents the average across the two sessions, the *y*-axis represents the difference between the two sessions, the solid horizontal bar indicates the mean difference across sessions, and the dashed horizontal lines indicate ±1.96 standard deviations from the mean. Good agreement is demonstrated when the mean difference is close to 0, there is an even spread of data points (each data point represents a single participant), and each data point falls within the dashed lines. Bland–Altman plots are especially useful for interpreting clinical significance even if the two sessions show statistical agreement, as this additional analysis helps to draw inferences regarding whether the difference between sessions is small enough for practical applications.

To further determine whether the prediction accuracy of the TRF model was reliable across sessions, a linear mixed-effect regression was run separately for each frequency band and stimulus feature (e.g., delta band broadband envelope, delta band broadband envelope derivative, theta band multiband envelope, etc.). Each model included prediction accuracy as the dependent variable and interactions of story by session and reference by session as predictors. Participant was included as a random intercept. This analysis was conducted separately for each electrode channel. The results at the Cz electrode are provided for brevity. The results for all electrodes are available at https://osf.io/pjau8/.

To rule out the possibility that ICC results were driven by noise across sessions, the influence of signal-to-noise ratio (SNR) on prediction accuracies across sessions was examined. SNR was estimated by taking the root mean square (RMS) of post-stimulus activity divided by the RMS of pre-stimulus activity. Two linear mixed-effect models with prediction accuracy as the outcome measure were compared: one included the interaction of stimulus, feature, frequency band, reference electrode, and session as predictors, and one included SNR as part of the interaction. To determine whether the inclusion of SNR improved model fit, ANOVA was used to compare the two models.

Lastly, considering that there were slight differences in sound intensity and age across participants, additional models were run to ensure that neither sound intensity nor age was a significant contributor to results. Linear mixed-effect models were run separately for each frequency band and stimulus feature (e.g., delta band broadband envelope, delta band broadband envelope derivative, theta band multiband envelope, etc.). To estimate sound intensity as precisely as possible, we took the RMS of each participant's StimTrak recording of the audio, then computed 10 times the log10 of the RMS (RMSlog). Each model was run separately for each electrode channel and included prediction accuracy as the dependent variable and interactions of story by session by RMSlog and reference by session by RMSlog as predictors. Participant was included as a random intercept. Neither the main effect of RMSlog nor any interaction including RMSlog was significant. Similarly, we ran each model with interactions of story by session by age and reference by session by age as predictors. Again, neither the main effect of age nor any interaction including age was significant.

*Data accessibility*. Downsampled data and fully preprocessed data (EEGLAB .set files) have been made publicly available via the Open Science Framework and can be accessed at https://osf.io/pjau8/. Raw data are available upon request.

## Results

**Figure 1.**
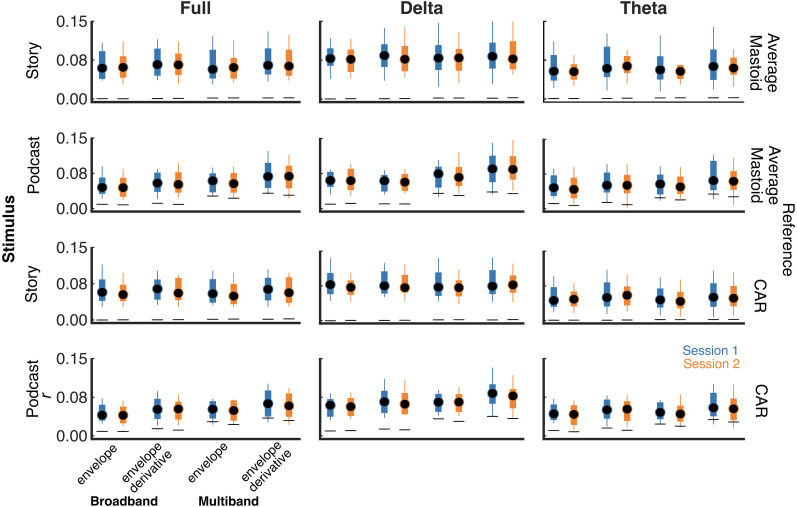
Boxplots of prediction accuracies for Session 1 and Session 2 for each stimulus (story, podcast), feature (broadband envelope and its derivative, multiband envelope and its derivative), frequency band (delta, theta, full), and reference electrode (average mastoid, CAR). Circles indicate the median across participants, the bottom and top bounds of the box reflect the first and third quartiles, and the whiskers reflect the range inclusive of the most extreme data points that are not outliers. The solid black lines represent mean chance performance.

Prediction accuracies of the TRF models are presented in Figure 1, averaged across electrodes. Figure 1 presents the median values across participants, the first and third quartiles, and the range inclusive of the most extreme data points that are not outliers. For each model, the median prediction accuracy across participants was better than the mean chance prediction accuracy across participants. We also note that, for each participant, the prediction accuracy for each model was better than the mean prediction accuracy for that participant's chance model. In other words, each participant's prediction accuracy for each session was better than the mean of their chance distribution.

Table 1 presents the results for channel Cz for the linear mixed-effect models with prediction accuracy as the dependent variable and interactions of story by session and reference by session as predictors for the different stimuli and features tested. Table 2 presents the ICCs for channel Cz prediction accuracies across sessions.

**Table 1.** Results of the linear mixed-effect models for channel Cz with prediction accuracy as the dependent variable and interactions of story by session and reference by session

	Stimulus		Session		Reference		Stimulus × Session		Reference × Session	
	*χ* ^2^	*p*	*χ* ^2^	*p*	*χ* ^2^	*p*	*χ* ^2^	*p*	*χ* ^2^	*p*
Full Band										
Broadband Envelope	0.385	0.535	0.000	0.991	4.520	0.034*	1.249	0.264	0.439	0.508
Broadband Envelope Derivative	0.310	0.578	0.077	0.782	3.551	0.059	2.319	0.128	0.307	0.580
Multiband Envelope	0.034	0.855	0.002	0.965	2.920	0.087	0.007	0.936	0.084	0.772
Multiband Envelope Derivative	6.272	0.012*	0.341	0.559	4.209	0.040*	1.663	0.197	0.380	0.538
Delta Band										
Broadband Envelope	0.219	0.640	0.000	0.995	2.705	0.100	0.121	0.728	0.134	0.715
Broadband Envelope Derivative	0.418	0.518	0.188	0.664	2.464	0.116	0.776	0.378	0.278	0.598
Multiband Envelope	0.275	0.600	0.002	0.968	1.834	0.176	0.234	0.629	0.044	0.833
Multiband Envelope Derivative	5.522	0.019*	0.027	0.869	2.021	0.155	0.034	0.854	0.041	0.839
Theta Band										
Broadband Envelope	0.488	0.485	0.114	0.735	4.462	0.035*	6.111	0.013*	0.810	0.368
Broadband Envelope Derivative	0.517	0.472	1.772	0.183	4.535	0.033*	6.403	0.011*	0.608	0.435
Multiband Envelope	0.297	0.586	0.088	0.766	3.785	0.052	0.487	0.485	0.156	0.693
Multiband Envelope Derivative	2.940	0.086	0.956	0.328	6.184	0.013*	4.764	0.029*	0.974	0.324

Significant effects are indicated with an asterisk.

**Table 2.** ICC results for prediction accuracies for channel Cz presented separately for each reference (CAR, average mastoid), frequency band (full, delta, theta), stimulus (story, podcast), and feature (broadband envelope and its derivative, multiband envelope, and its derivative)

		Broadband Envelope				Broadband Envelope Derivative				Multiband Envelope				Multiband Envelope Derivative			
		Story		Podcast		Story		Podcast		Story		Podcast		Story		Podcast	
		ICC	*p*	ICC	*p*	ICC	*p*	ICC	*p*	ICC	*p*	ICC	*p*	ICC	*p*	ICC	*p*
CAR	Full Band	0.71	0.00	0.56	0.00	0.78	0.00	0.52	0.00	0.57	0.00	0.51	0.00	0.71	0.00	0.63	0.00
	Delta Band	0.50	0.00	0.35	0.04	0.72	0.00	0.39	0.02	0.43	0.02	0.29	0.07	0.62	0.00	0.44	0.01
	Theta Band	0.66	0.00	0.46	0.01	0.74	0.00	0.60	0.00	0.66	0.00	0.71	0.00	0.70	0.00	0.80	0.00
Average Mastoid	Full Band	0.69	0.00	0.66	0.00	0.81	0.00	0.51	0.00	0.63	0.00	0.62	0.00	0.78	0.00	0.74	0.00
	Delta Band	0.58	0.00	0.52	0.00	0.72	0.00	0.36	0.03	0.45	0.01	0.43	0.01	0.66	0.00	0.63	0.00
	Theta Band	0.74	0.00	0.64	0.00	0.84	0.00	0.63	0.00	0.81	0.00	0.74	0.00	0.83	0.00	0.78	0.00

### Broadband envelope and derivative

#### Full band

**Figure 2.**
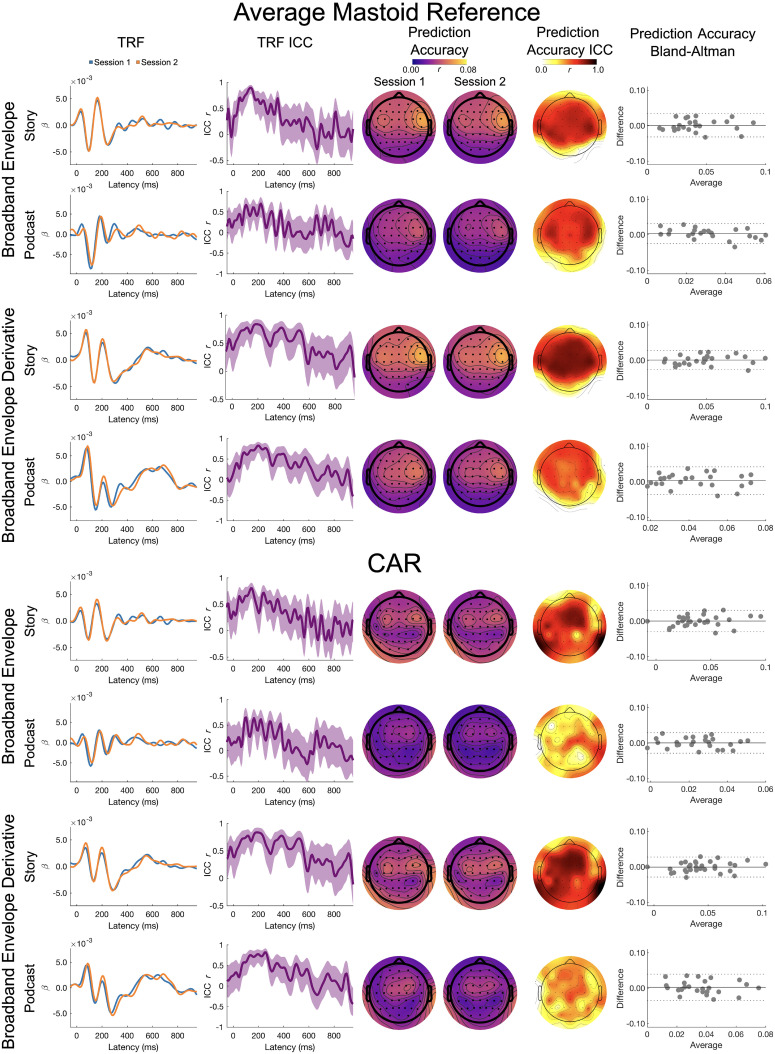
Full band: broadband envelope and broadband envelope derivative. The TRF column presents the TRFs for Session 1 (blue) and Session 2 (orange), and the TRF ICC column presents the ICC for the Session 1 and Session 2 TRFs; shading denotes the 95% confidence interval of the ICC across participants. The prediction accuracy column presents topoplots of prediction accuracies for Session 1 and Session 2, and the prediction accuracy ICC column presents the topoplot of the ICCs on these prediction accuracies. The prediction accuracy Bland–Altman column presents Bland–Altman plots for the Session 1 and Session 2 prediction accuracies, where the *x*-axis is the average across Sessions 1 and 2, the *y*-axis is the difference between Sessions 1 and 2, the solid horizontal line is the mean difference, and the dashed horizontal lines are ±1.96 standard deviations from the mean; each dot represents a participant. The top four rows are the average mastoid reference, and the bottom four rows are the CAR. Data presented for channel Cz.

Full band results for the broadband envelope and its derivative are presented in Figure 2.

*TRF ICC*. On visual inspection, the TRFs for the story showed a moderate to high degree of similarity across sessions. The ICCs for the TRFs were the largest between 50 and 300 ms for the broadband envelope for the average mastoid (ICC ∼0.5 to ∼0.9), with slightly lower similarity for the CAR (ICC ∼0.5 to ∼0.75). For the broadband envelope derivative, the ICCs were highest between 50 and 500 ms for the average mastoid (ICC ∼0.4 to ∼0.8) and CAR (ICC ∼0.4 to ∼0.75).

Visual inspection of the TRFs for the podcast showed a moderate degree of similarity across sessions. The ICCs for the TRFs were the largest between 100 and 250 ms (ICC ∼0.4 to ∼0.6) for the broadband envelope for both reference schemes. The ICCs were slightly higher for the broadband envelope derivative, with the highest ICCs between 150 and 350 ms for both reference schemes (ICC ∼0.5 to ∼0.8).

*Prediction accuracy LMEM*. For the broadband envelope, there was no significant main effect of story or session. The reference however showed a small but significant main effect (bbenv: *η*_p_^2^ = 0.025) reflecting higher prediction accuracies for the average mastoid reference (bbenv emmean = 0.038) than CAR (bbenv emmean = 0.029). The interaction effects were not significant. This indicates that the prediction accuracies were stable across sessions but changed with the choice of reference. Unlike the broadband envelope, for the broadband envelope derivative, none of the effects were significant predictors of prediction accuracy.

*Prediction accuracy ICC and Bland–Altman*. The ICC for the average mastoid reference showed moderate to good test–retest reliability for both the broadband envelope (Albert, ICC_Cz_ = 0.692; *p* < 0.001; Yankees, ICC_Cz_ = 0.660; *p* < 0.001) and its derivative (Albert, ICC_Cz_ = 0.815; *p* < 0.001; Yankees, ICC_Cz_ = 0.507; *p* = 0.003). The largest ICCs were seen in frontocentral and frontotemporal channels. The Bland–Altman plots also show stable prediction accuracies with low spread of differences across sessions and tight limits of agreement for both the stimuli.

The ICC for the CAR showed poor to good test–retest reliability for both the broadband envelope (Albert, ICC_Cz_ = 0.714; *p* < 0.001; Yankees, ICC_Cz_ = 0.564; *p* = 0.001) and its derivative (Albert, ICC_Cz_ = 0.775; *p* < 0.001; Yankees, ICC_Cz_ = 0.389; *p* = 0.022) at Cz. While most frontocentral channels showed the largest ICCs for the story, only a few central electrodes showed strong ICCs and the majority of the channels did not show strong ICCs. However, such moderate reliability was only seen in very few frontocentral channels for the podcast suggesting lower reliability. It is worth noting that the Bland–Altman plot shows stable prediction accuracies with low spread of differences across sessions and tight limits of agreements for both stimuli. However, the same was not true for the podcast in channels other than Cz.

#### Delta band

**Figure 3.**
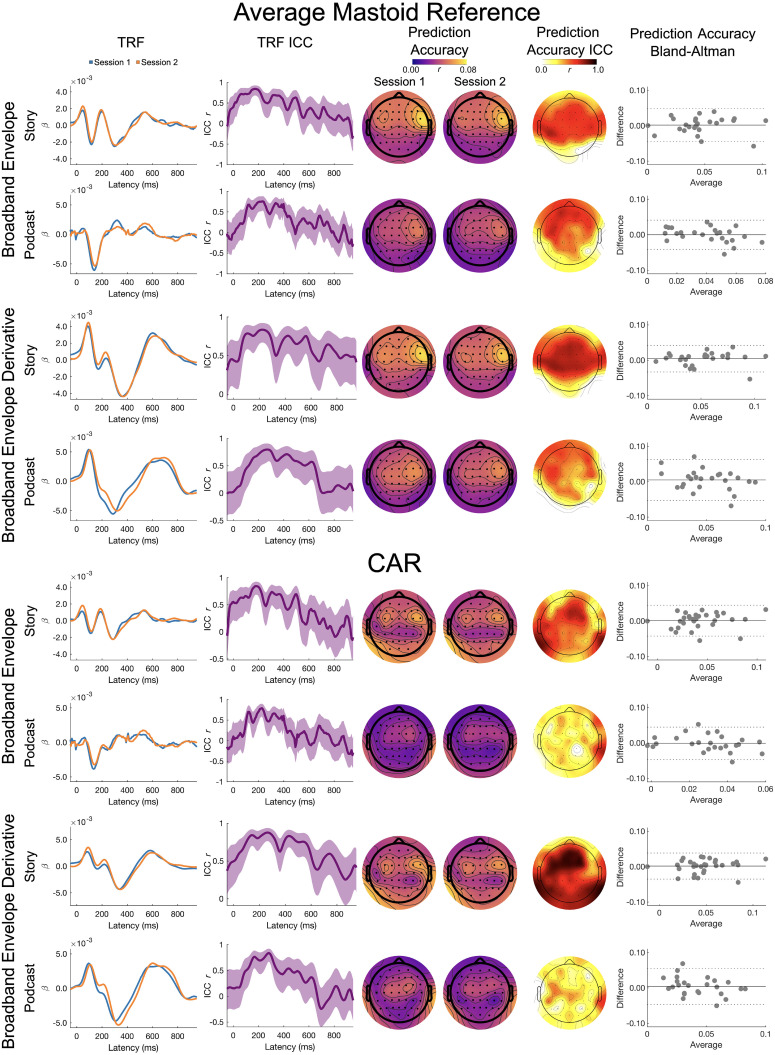
Delta band: broadband envelope and broadband envelope derivative. The TRF column presents the TRFs for Session 1 (blue) and Session 2 (orange), and the TRF ICC column presents the ICC for the Session 1 and Session 2 TRFs; shading denotes the 95% confidence interval of the ICC. The prediction accuracy column presents topoplots of prediction accuracies for Session 1 and Session 2, and the prediction accuracy ICC column presents the topoplot of the ICCs on these prediction accuracies. The prediction accuracy Bland–Altman column presents Bland–Altman plots for the Session 1 and Session 2 prediction accuracies, where the *x*-axis is the average across Sessions 1 and 2, the *y*-axis is the difference between Sessions 1 and 2, the solid horizontal line is the mean difference, and the dashed horizontal lines are ±1.96 standard deviations from the mean; each dot represents a participant. The top four rows are the average mastoid reference, and the bottom four rows are the CAR. Data presented for channel Cz.

Delta band results for the broadband envelope and its derivative are presented in Figure 3.

*TRF ICC*. On visual inspection, the TRFs for the story showed a moderate to high degree of similarity. For the story, the ICCs for the TRFs were the largest between 50 and 400 ms for the broadband envelope for the average mastoid (ICC ∼0.5 to ∼0.9), with slightly lower ICCs for the CAR (ICC ∼0.5 to ∼0.8). For the broadband envelope derivative, the ICCs for the story were the highest between 50 and 600 ms for both reference schemes (ICC ∼0.4 to ∼0.75).

Visual inspection of the TRFs for the podcast also showed a moderate to high degree of similarity across sessions. The ICCs for the TRFs were the largest between 100 and 400 ms for the broadband envelope in both the reference schemes (ICC ∼0.5 to ∼0.75). However, for the broadband envelope derivative, differences were observed between the two reference schemes. ICCs were highest between 150 and 500 ms for the average mastoid reference (ICC ∼ 0.5 to ∼0.75), with slightly higher similarity observed for the CAR between 100 and 400 ms (ICC ∼0.5 to ∼0.90).

*Prediction accuracy LMEM*. There were no significant main effects of story, session, or interactions for the prediction accuracies using both the broadband envelope and broadband envelope derivatives. This suggests that the prediction accuracies did not differ across sessions and were not affected by the choice of story or reference in the delta band for these two stimulus features.

*Prediction accuracy ICC and Bland–Altman*. The ICC for the average mastoid reference showed poor to moderate test–retest reliability for both the broadband envelope (Albert, ICC_Cz_ = 0.583; *p* < 0.001; Yankees, ICC_Cz_ = 0.522; *p* = 0.003) and its derivative (Albert, ICC_Cz_ = 0.719; *p* < 0.001; Yankees, ICC_Cz_ = 0.364; *p* = 0.030). The largest ICCs were seen in frontocentral and frontotemporal channels. The Bland–Altman plots also showed stable prediction accuracies with low spread of differences across sessions and tight limits of agreement for both stimuli (Fig. 3), though there were two outliers.

The ICC for the CAR showed poor to moderate test–retest reliability for both the broadband envelope (Albert, ICC_Cz_ = 0.497; *p* = 0.005; Yankees, ICC_Cz_ = 0.346; *p* = 0.043) and its derivative (Albert, ICC_Cz_ = 0.721; *p* < 0.001; Yankees, ICC_Cz_ = 0.518; *p* = 0.003). The Bland–Altman plots also showed stable prediction accuracies with low spread of differences across sessions and tight limits of agreement for both stimuli, though there were two outliers.

#### Theta band

**Figure 4.**
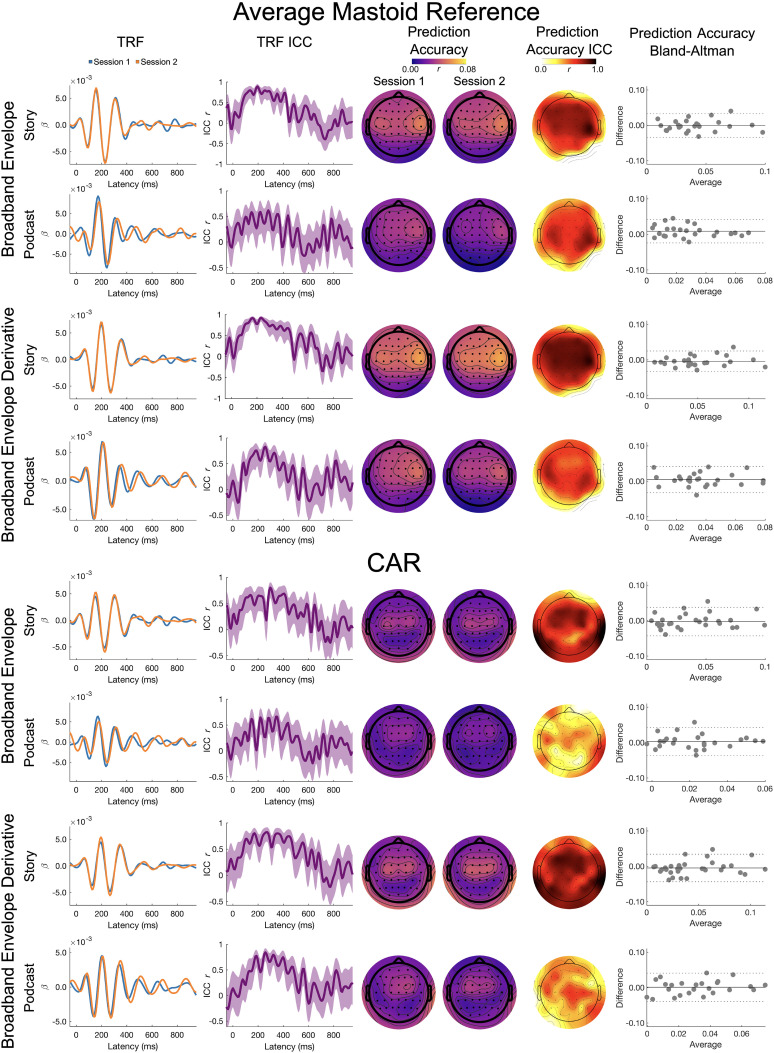
Theta band: broadband envelope and broadband envelope derivative. The TRF column presents the TRFs for Session 1 (blue) and Session 2 (orange), and the TRF ICC column presents the ICC for the Session 1 and Session 2 TRFs; shading denotes the 95% confidence interval of the ICC. The prediction accuracy column presents topoplots of prediction accuracies for Session 1 and Session 2, and the prediction accuracy ICC column presents the topoplot of the ICCs on these prediction accuracies. The prediction accuracy Bland–Altman column presents Bland–Altman plots for the Session 1 and Session 2 prediction accuracies, where the *x*-axis is the average across Sessions 1 and 2, the *y*-axis is the difference between Sessions 1 and 2, the solid horizontal line is the mean difference, and the dashed horizontal lines are ±1.96 standard deviations from the mean; each dot represents a participant. The top four rows are the average mastoid reference and the bottom four rows are the CAR. Data presented for channel Cz.

Theta band results for the broadband envelope and its derivative are presented in Figure 4.

*TRF ICC*. On visual inspection, the TRFs for the story showed a moderate to high degree of similarity. The ICCs for the TRFs were the largest for the broadband envelope between 100 and 400 ms, with slightly higher similarity for the average mastoid reference (ICC ∼0.5 to ∼0.9) than CAR (ICC ∼0.4 to ∼0.75). For the broadband envelope derivative, the ICCs for the TRFs were similar for the average mastoid and CAR reference schemes, with the largest ICCs between 100 and 425 ms (ICC ∼0.5 to 0.75).

Visual inspection of the TRFs to the podcast differed slightly across sessions, showing poor to moderate degrees of similarity. For the broadband envelope, the ICCs for the TRFs were highest between 100 and 350 ms, with slightly higher similarity for the average mastoid reference (ICC ∼0.25 to ∼0.60) than the CAR (ICC ∼0.0 to ∼0.60). For the broadband envelope derivative, the ICCs for the TRFs were highest between 100 and 350 ms for the average mastoid reference (ICC ∼0.40 to ∼0.75) and between 100 and 450 ms for the CAR (ICC ∼0.50 to ∼0.75).

*Prediction accuracy LMEM*. There were no significant main effects of story or session nor a significant interaction of reference and session. However, there was a small but statistically significant main effect of reference (bbenv, *η_p_*^2^ = 0.025; bbenv deriv, *η_p_*^2^ = 0.025), where prediction accuracies were higher for average mastoid reference (bbenv emmean = 0.036; bbenv deriv emmean = 0.045) than CAR (bbenv emmean = 0.028; bbenv deriv emmean = 0.036). There was also a significant interaction of story by session (bbenv, *η_p_*^2^ = 0.033; bbenv, *η_p_*^2^ = 0.035), which primarily reflects a significant difference in prediction accuracies between the Yankees podcast and the Albert story in Session 2 (Yankees bbenv emmean Session 2 = 0.023; Albert bbenv emmean Session 2 = 0.039; Yankees bbenv deriv emmean Session 2 = 0.033; Albert bbenv deriv emmean Session 2 = 0.049) but not Session 1 (Yankees bbenv emmean session 1 = 0.0300; Albert bbenv emmean Session 1 = 0.036; Yankees bbenv deriv emmean Session 2 = 0.037; Albert bbenv deriv emmean Session 2 = 0.043).

*Prediction accuracy ICC and Bland–Altman*. The ICC for the average mastoid reference showed good test–retest reliability for both the broadband envelope (Albert, ICC_Cz_ = 0.744; *p* < 0.001; Yankees, ICC_Cz_ = 0.639; *p* < 0.001) and its derivative (Albert, ICC_Cz_ = 0.839; *p* < 0.001; Yankees, ICC_Cz_ = 0.631; *p* < 0.001).

The ICC for the CAR showed poor to good test–retest reliability for both the broadband envelope (Albert, ICC_Cz_ = 0.662; *p* < 0.001; Yankees, ICC_Cz_ = 0.464; *p* = 0.007) and its derivative (Albert, ICC_Cz_ = 0.737; *p* < 0.001; Yankees, ICC_Cz_ = 0.600; *p* < 0.001).

### Multiband envelope and derivative

#### Full band

**Figure 5.**
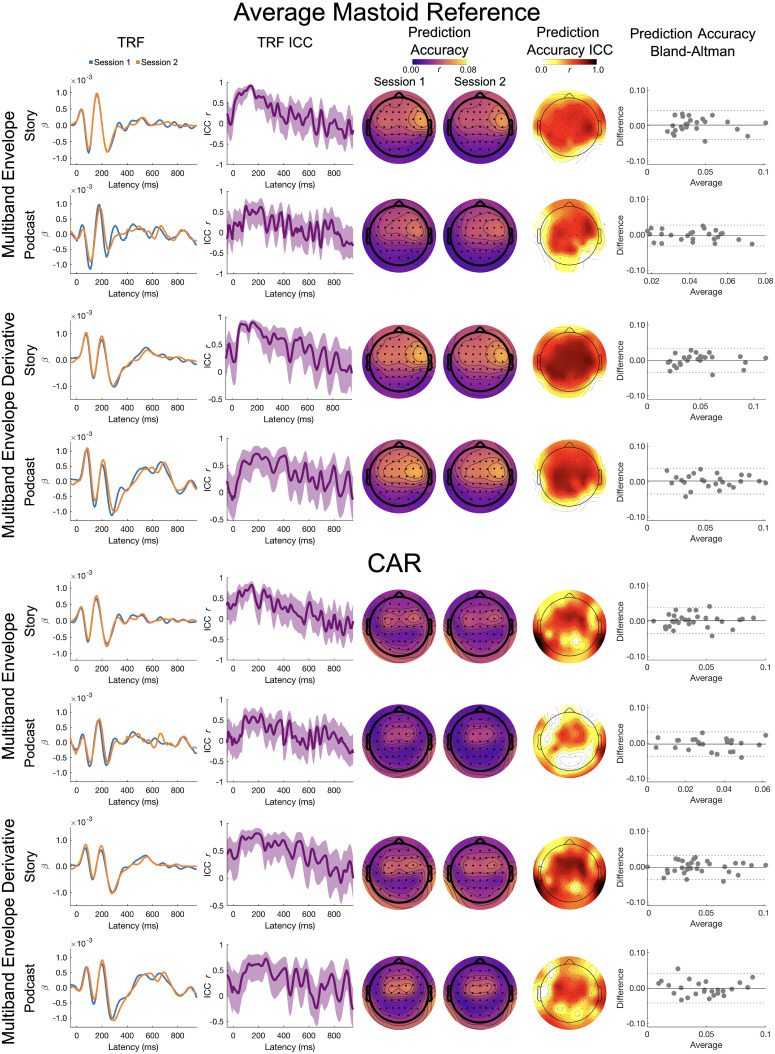
Full band: multiband envelope and multiband envelope derivative. The TRF column presents the TRFs for Session 1 (blue) and Session 2 (orange), and the TRF ICC column presents the ICC for the Session 1 and Session 2 TRFs; shading denotes the 95% confidence interval of the ICC. The prediction accuracy column presents topoplots of prediction accuracies for Session 1 and Session 2, and the prediction accuracy ICC column presents the topoplot of the ICCs on these prediction accuracies. The prediction accuracy Bland–Altman column presents Bland–Altman plots for the Session 1 and Session 2 prediction accuracies, where the *x*-axis is the average across Sessions 1 and 2, the *y*-axis is the difference between Sessions 1 and 2, the solid horizontal line is the mean difference, and the dashed horizontal lines are ±1.96 standard deviations from the mean; each dot represents a participant. The top four rows are the average mastoid reference, and the bottom four rows are the CAR. Data presented for channel Cz.

Full band results for the multiband envelope and its derivative are presented in Figure 5.

*TRF ICC*. On visual inspection, the TRFs for the story showed a moderate to high degree of similarity. The ICCs for the TRFs for the average mastoid reference were similar across the multiband envelope and multiband envelope derivative, with the largest ICCs between 50 and 300 ms (ICC ∼0.50 to ∼0.90). For the CAR, the ICCs for the TRFs were highest between 50 and 300 ms for the multiband envelope (ICC ∼0.25 to ∼0.75). The multiband envelope derivative ICCs were slightly higher, with the largest ICCs between 50 and 400 ms (ICC ∼0.50 to 0.75).

Visual inspection of the TRFs to the podcast showed a moderate degree of similarity across sessions. For the multiband envelope, the ICCs for the TRFs were highest between 100 and 225 ms and did not differ across reference schemes (ICC ∼0.50 to ∼0.60). For the multiband envelope derivative, the ICCs for the TRFs were highest between 50 and 400 ms for the average mastoid reference (ICC ∼0.40 to ∼0.70) and between 100 and 400 ms for the CAR (ICC ∼0.40 to ∼0.70).

*Prediction accuracy LMEM*. For the multiband envelope derivative, there was a small but significant main effect of reference (mbenv deriv, *η_p_*^2^ = 0.023), reflecting slightly higher prediction accuracies for the average mastoid reference (mbenv deriv emmean = 0.053) than CAR (mbenv deriv emmean = 0.044) but no effect of session or interaction of session with story or reference. For the multiband envelope derivative only, there was a small but significant main effect of story (mbenv deriv, *η_p_*^2^ = 0.034), reflecting slightly higher prediction accuracies for the Yankees podcast (mbenv deriv emmean = 0.052) than the Albert story (mbenv deriv emmean = 0.045).

*Prediction accuracy ICC and Bland–Altman*. The ICC for the average mastoid reference showed moderate to good test–retest reliability for both the multiband envelope (Albert, ICC_Cz_ = 0.633; *p* < 0.001; Yankees, ICC_Cz_ = 0.617; *p* < 0.001) and its derivative (Albert, ICC_Cz_ = 0.783; *p* < 0.001; Yankees, ICC_Cz_ = 0.737; *p* < 0.001).

The ICC for the CAR showed moderate test–retest reliability for both the multiband envelope (Albert, ICC_Cz_ = 0.570; *p* = 0.001; Yankees, ICC_Cz_ = 0.506; *p* = 0.004) and its derivative (Albert, ICC_Cz_ = 0.705; *p* < 0.001; Yankees, ICC_Cz_ = 0.631; *p* < 0.001).

#### Delta band

**Figure 6.**
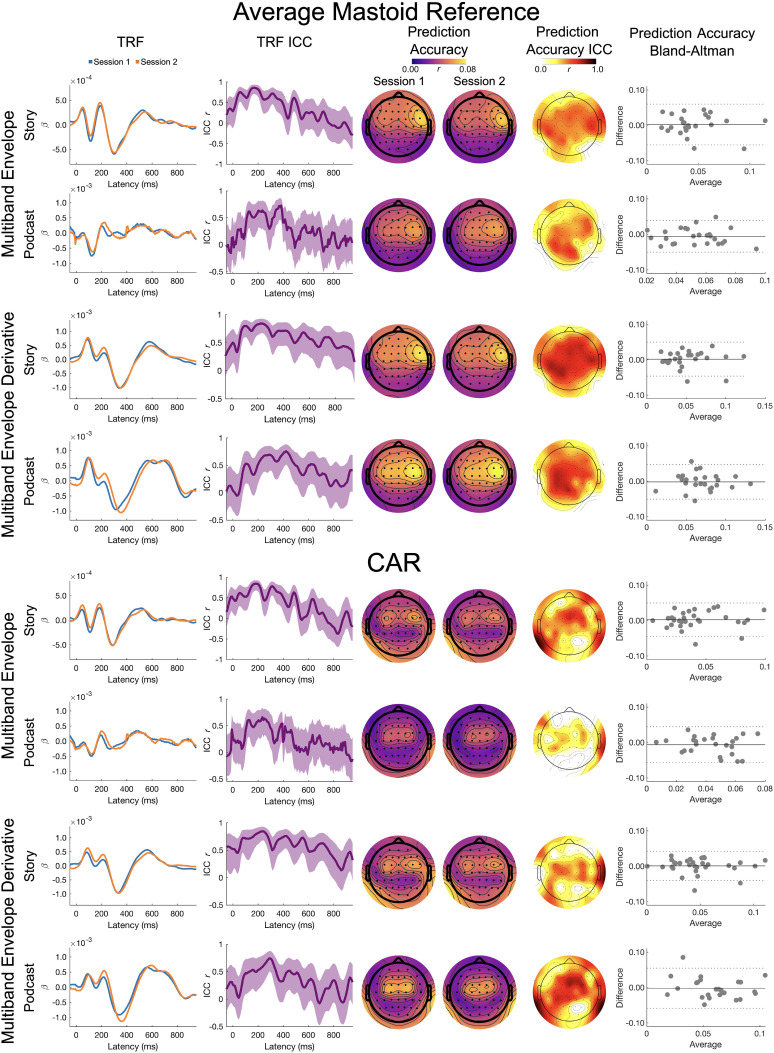
Delta band: multiband envelope and multiband envelope derivative. The TRF column presents the TRFs for Session 1 (blue) and Session 2 (orange), and the TRF ICC column presents the ICC for the Session 1 and Session 2 TRFs; shading denotes the 95% confidence interval of the ICC. The prediction accuracy column presents topoplots of prediction accuracies for Session 1 and Session 2, and the prediction accuracy ICC column presents the topoplot of the ICCs on these prediction accuracies. The prediction accuracy Bland–Altman column presents Bland–Altman plots for the Session 1 and Session 2 prediction accuracies, where the *x*-axis is the average across Sessions 1 and 2, the *y*-axis is the difference between Sessions 1 and 2, the solid horizontal line is the mean difference, and the dashed horizontal lines are ±1.96 standard deviations from the mean; each dot represents a participant. The top four rows are the average mastoid reference, and the bottom four rows are the CAR. Data presented for channel Cz.

Delta band results for the multiband envelope and its derivative are presented in Figure 6.

*TRF ICC*. On visual inspection, the TRFs for the story showed a moderate to high degree of similarity across sessions. The ICCs for the TRFs for the multiband envelope were similar across the reference schemes (ICC ∼0.50 to ∼0.75), with the largest ICCs between 25 and 400 ms for the average mastoid and between 25 and 600 ms for the CAR. For the multiband envelope derivative, the ICCs for the TRFs were highest between 25 and 400 ms for the average mastoid (ICC ∼0.50 to ∼0.90). For the CAR, the ICCs were slightly lower, with the highest ICCs between 50 and 600 ms (ICC ∼0.50 to ∼0.80).

Visual inspection of the TRFs to the podcast showed a moderate degree of similarity across sessions. For the multiband envelope, the ICCs for the TRFs were highest between 100 and 400 ms for the average mastoid reference (ICC ∼0.25 to ∼0.75) and between 100 and 700 ms for the CAR (ICC ∼0.30 to ∼0.75). For the multiband envelope derivative, the ICCs for the TRFs were highest between 100 and 300 ms for the average mastoid reference (ICC ∼0.50 to ∼0.60), with slightly higher ICCs observed between 100 and 300 ms for the CAR (ICC ∼0.50 to ∼0.75).

*Prediction accuracy LMEM*. A small but significant main effect of story was observed for the multiband envelope derivative (mbenv deriv, *η_p_*^2^ = 0.030), reflecting slightly higher prediction accuracies for the Yankees podcast (mbenv deriv emmean = 0.066) than the Albert story (mbenv deriv emmean = 0.049). No other significant effects were observed in the multiband envelope or multiband envelope derivative.

*ICC and Bland–Altman*. The ICC for the average mastoid reference showed poor to moderate test–retest reliability for both the multiband envelope (Albert, ICC_Cz_ = 0.451; *p* = 0.012; Yankees, ICC_Cz_ = 0.427; *p* = 0.012) and its derivative (Albert, ICC_Cz_ = 0.663; *p* < 0.001; Yankees, ICC_Cz_ = 0.630; *p* < 0.001).

The ICC for the CAR showed poor to moderate test–retest reliability for both the multiband envelope (Albert, ICC_Cz_ = 0.425; *p* = 0.017; Yankees, ICC_Cz_ = 0.285; *p* = 0.073) and its derivative (Albert, ICC_Cz_ = 0.621; *p* < 0.001; Yankees, ICC_Cz_ = 0.439; *p* = 0.014).

#### Theta band

**Figure 7.**
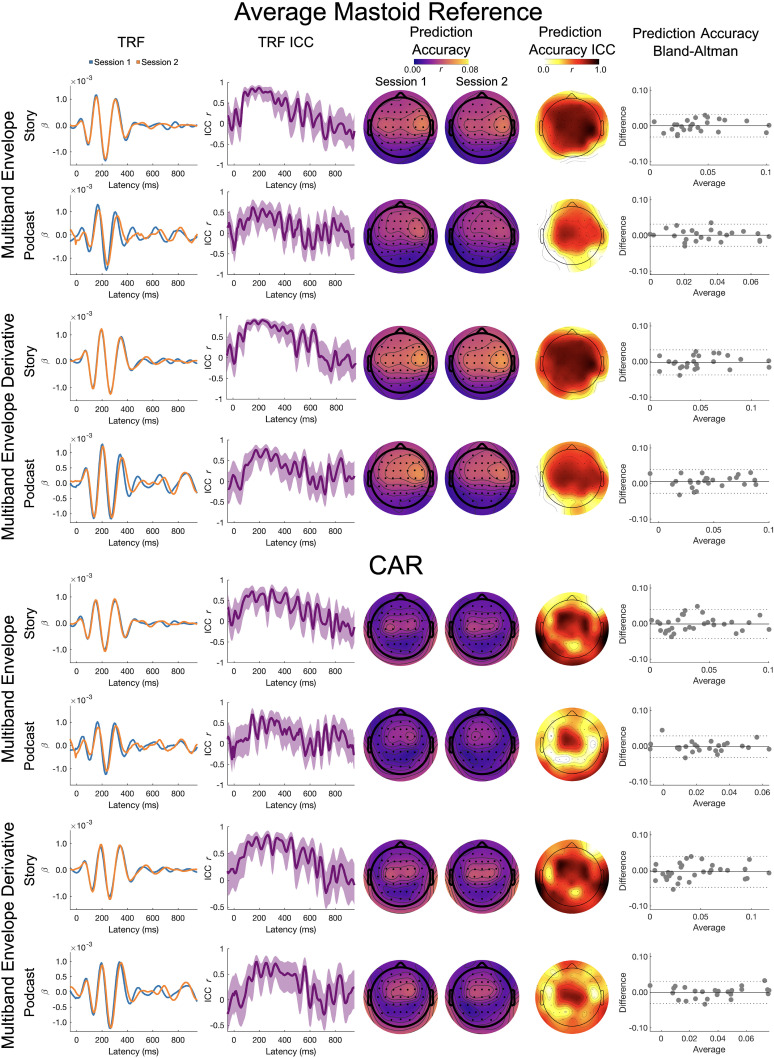
Theta band: multiband envelope and multiband envelope derivative. The TRF column presents the TRFs for Session 1 (blue) and Session 2 (orange), and the TRF ICC column presents the ICC for the Session 1 and Session 2 TRFs; shading denotes the 95% confidence interval of the ICC. The prediction accuracy column presents topoplots of prediction accuracies for Session 1 and Session 2, and the prediction accuracy ICC column presents the topoplot of the ICCs on these prediction accuracies. The prediction accuracy Bland–Altman column presents Bland–Altman plots for the Session 1 and Session 2 prediction accuracies, where the *x*-axis is the average across Sessions 1 and 2, the *y*-axis is the difference between Sessions 1 and 2, the solid horizontal line is the mean difference, and the dashed horizontal lines are ±1.96 standard deviations from the mean; each dot represents a participant. The top four rows are the average mastoid reference, and the bottom four rows are the CAR. Data presented for channel Cz.

Theta band results for the multiband envelope and its derivative are presented in Figure 7.

*TRF ICC*. On visual inspection, the TRFs for the story showed a poor to high degree of similarity across sessions. The ICCs for the TRFs for the multiband envelope were largest for the average mastoid reference between 50 and 450 ms (ICC ∼0.50 to ∼0.90), with lower ICCs for the CAR between 100 and 450 ms (ICC ∼0.25 to ∼0.75). For the multiband envelope derivative, the ICCs for the TRFs were highest between 100 and 425 ms for the average mastoid (ICC ∼0.50 to ∼0.90), with slightly lower ICCs for the CAR between 100 and 450 ms (ICC ∼0.50 to ∼0.80).

Visual inspection of the TRFs to the podcast showed a poor to moderate degree of similarity across sessions. For the multiband envelope, the ICCs for the TRFs were highest between 100 and 375 ms for the average mastoid reference (ICC ∼0.25 to ∼0.60), with slightly higher ICCs for the CAR observed between 100 and 500 ms (ICC ∼0.25 to ∼0.75). For the multiband envelope derivative, the ICCs for the TRFs were highest between 100 and 300 ms for the average mastoid reference (ICC ∼0.50 to ∼0.75) and between 100 and 500 ms for the CAR (ICC ∼0.40 to ∼0.75).

*LMEM*. There were no significant main effects or interactions observed for the multiband envelope. For the multiband envelope derivative, there was a small but significant main effect of reference (mbenv deriv, *η_p_*^2^ = 0.034), reflecting slightly higher prediction accuracies for the average mastoid reference (mbenv deriv emmean = 0.049) than CAR (mbenv deriv emmean = 0.039). There was also a significant interaction of story by session (mbenv deriv, *η_p_*^2^ = 0.026), although post hoc comparisons were not significant after Tukey's adjustment. No other comparisons were significant for the multiband envelope derivative.

*ICC and Bland–Altman*. The ICC for the average mastoid reference showed moderate to good test–retest reliability for both the multiband envelope (Albert, ICC_Cz_ = 0.814; *p* < 0.001; Yankees, ICC_Cz_ = 0.738; *p* < 0.001) and its derivative (Albert, ICC_Cz_ = 0.827; *p* < 0.001; Yankees, ICC_Cz_ = 0.785; *p* < 0.001).

The ICC for the CAR showed moderate to good test–retest reliability for both the multiband envelope (Albert, ICC_Cz_ = 0.659; *p* < 0.001; Yankees, ICC_Cz_ = 0.715; *p* < 0.001) and its derivative (Albert, ICC_Cz_ = 0.701; *p* < 0.001; Yankees, ICC_Cz_ = 0.801; *p* < 0.001).

### Test–retest reliability estimates for varying amounts of data

**Figure 8.**
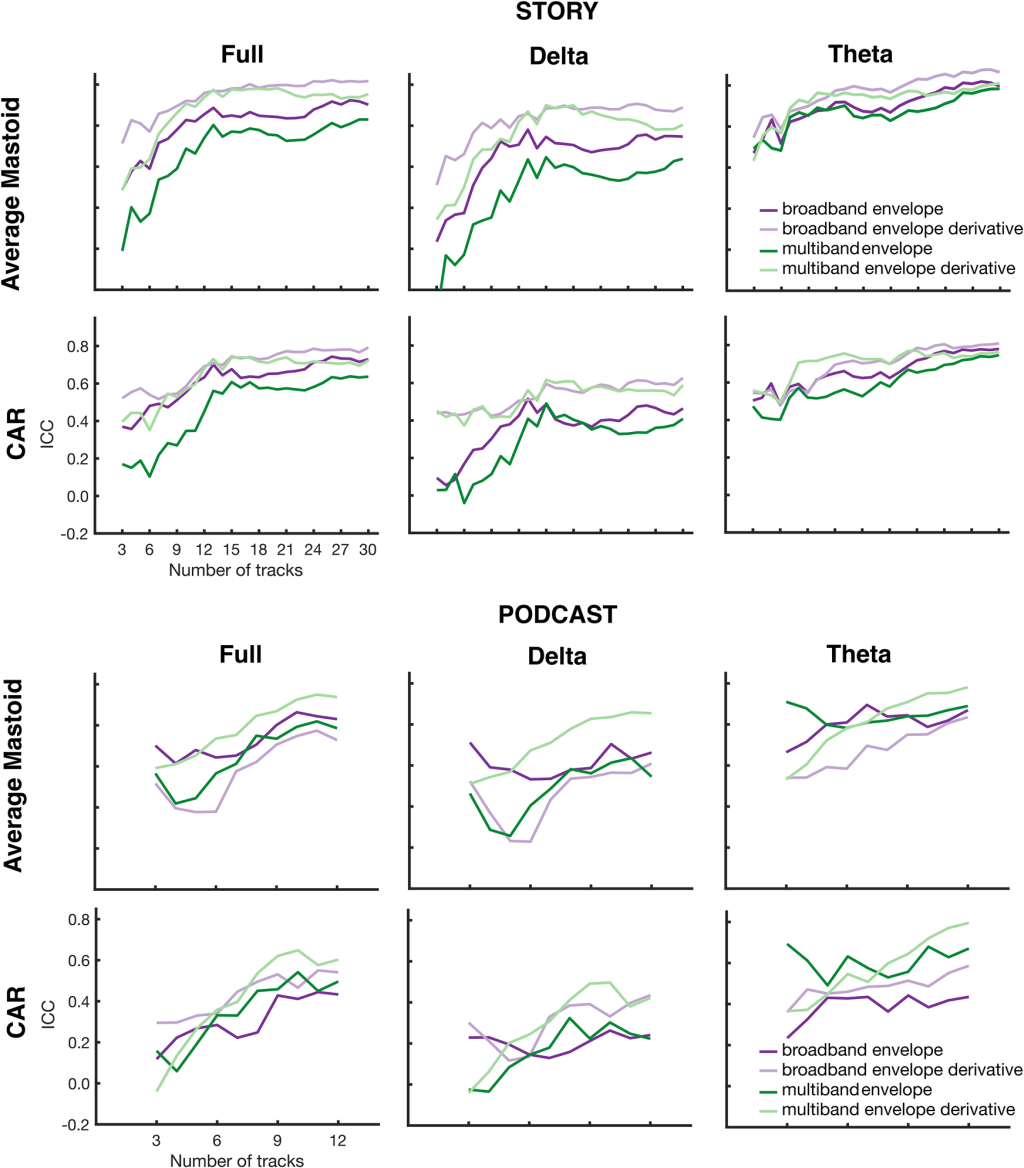
ICCs on prediction accuracy for the audiobook (story) and podcast for each frequency band (full, delta, theta) for each reference scheme (average mastoid, CAR) with different amounts of data. The number of 65 s tracks used to estimate the TRF is on the *x*-axis, and ICCs on the resulting prediction accuracies are on the *y*-axis. The color of the lines reflect different stimulus features (dark purple = broadband envelope; light purple = broadband envelope derivative; dark green = multiband envelope; light green = multiband envelope derivative).

To determine the amount of data required before stable ICCs are reached, we evaluated the ICCs for prediction accuracy for 3–30 (audiobook) or 3–12 (podcast) 65-s-long segments of data. Figure 8 displays the resulting ICCs on the *y*-axis and number of segments on the *x*-axis separately for each story and frequency band. Across all frequency bands, the ICC increased with the amount of data before reaching a plateau. ICCs for the theta bands showed the least change with data, suggesting that data from as little as six segments (390 s) was sufficient for strong, stable ICCs. The ICC in the full band and the delta band, however, continued to increase until ∼12 segments (780 s). These patterns were true for both the story and the podcast across all stimulus features and both reference schemes. While the data across all features tended to have similar trends, the multiband features tended to show a slightly steeper change in ICCs, suggesting somewhat greater reliance on the amount of data.

#### SNR

**Figure 9.**
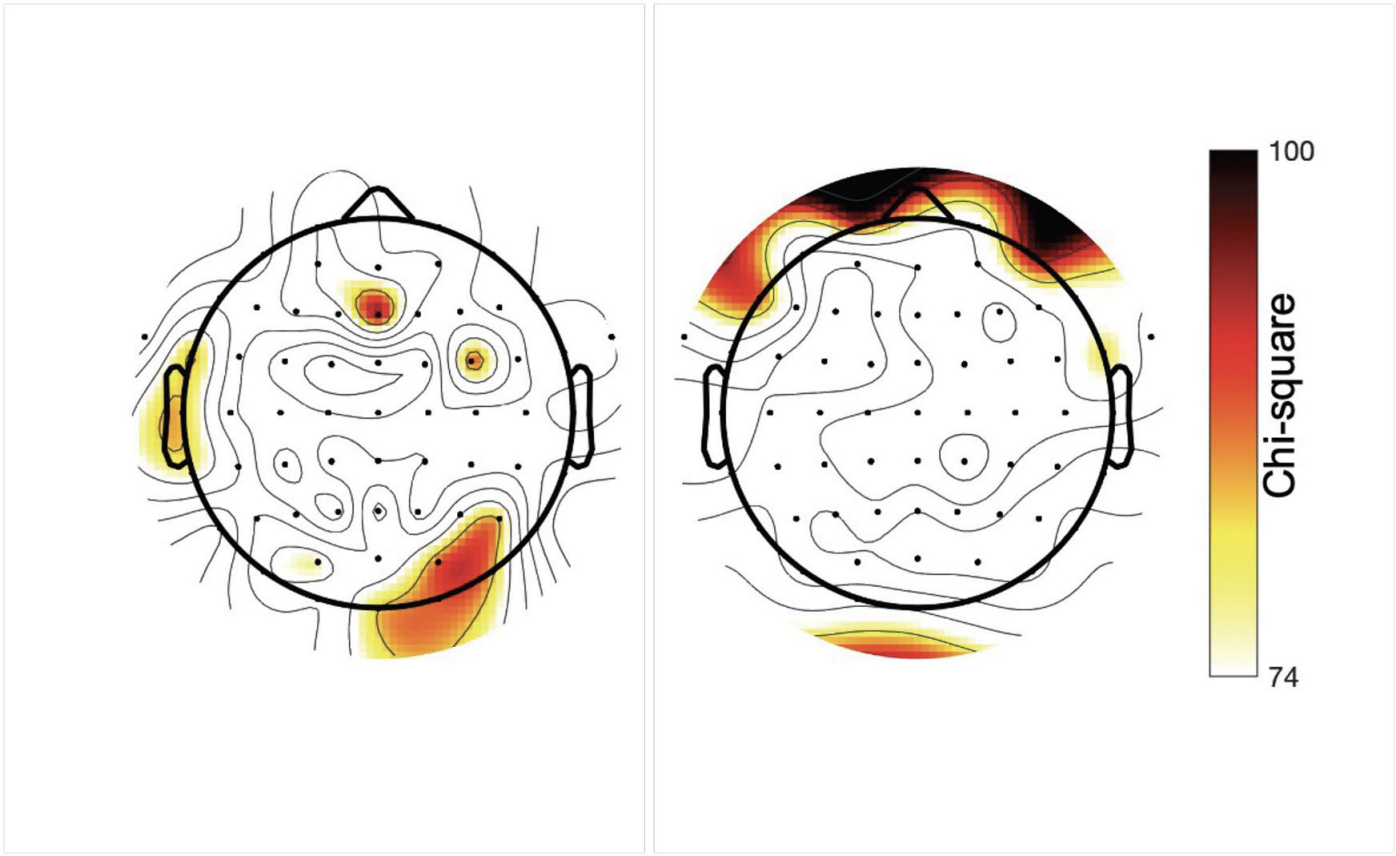
Results of ANOVA comparing linear mixed-effect models with and without the inclusion of SNR as a predictor. The left panel is the average mastoid reference, and the right panel is the CAR. *χ*^2^ is plotted, thresholded at FDR-corrected *p* < 0.05. White indicates that the electrode was not influenced by SNR across sessions.

For channel Cz, the ANOVA indicated that the addition of SNR to the model did not significantly improve model fit for the average mastoid reference (*χ*^2^_(48)_ = 58.00; *p* = 0.153) or the CAR (*χ*^2^_(48)_ = 34.07; *p* = 0.936). Figure 9 displays the results for each electrode channel for the ANOVA comparing models with and without SNR for the average mastoid reference and CAR, thresholded at FDR-corrected *p* < 0.05. As can be seen in Figure 9, SNR was a significant contributor to prediction accuracies for only a small subset of electrodes, primarily in frontal and occipital regions. This suggests that SNR should be considered when examining test–retest using the TRF approach, especially in noisy electrodes. Including SNR as a covariate in statistical analyses might help.

## Discussion

We investigated test–retest reliability of cortical tracking of the speech envelope in neurotypical adults, demonstrating moderate to good reliability and supporting its potential as a robust measure of auditory processing. Previous research reported similar ICCs for test–retest reliability (ICCs ∼0.40 to 0.90, with most between 0.6 and 0.85, Panela et al., 2024), albeit for a single stimulus feature and EEG frequency band. We built on previous work by examining test–retest reliability using two stimuli (audiobook, podcast), four stimulus features (broadband envelope and its derivative, multiband envelope, and its derivative), three EEG frequency bands (delta, theta, full), and two EEG references (average mastoid and CAR) to determine how cortical tracking metrics vary across these contrasts. Our selections were motivated by the literature, where researchers often use an audiobook or podcast, examine the envelope or its derivative, examine delta and theta bands (or sometimes a combined delta–theta band, here referred to as the full band), and use either the average mastoid or CAR. Essentially, we queried how these different methodological choices influence reliability of cortical tracking measures. We also examined the amount of data required for stable ICCs to be reached and the influence of SNR on test–retest reliability.

Our results show slightly higher test–retest reliability than Panela et al., in some instances even showing better reliability than their noise burst stimuli. It is worth noting that the ICCs observed in the current study are comparable to other neurophysiological measures in neurotypical and clinical populations (e.g., ICCs from 0.55 to 0.88 for resting-state EEG in stroke-induced aphasia and neurotypical controls; Dalton et al., 2021). ICCs of 0.7–0.8 are common across clinical measures that assess the neurophysiology of auditory processing (Bidelman et al., 2018) and are generally considered to be reliable in patient-related outcome measures (Nunnally and Bernstein, 1994; Frost et al., 2007) and acceptable for clinical use (Cicchetti, 1994). The current study also indicates that SNR was not a strong contributor to reliability and that stable ICCs can be reached with as little as 390 s of data for the theta band and 780 s for the delta and full bands. This finding complements recent work indicating that better-than-chance prediction accuracies for cortical tracking of the envelope can be achieved with ∼760 s (12 min) of data (Mesik and Wojtczak, 2023). Notably, that study examined tracking of the log-transformed speech envelope in the full band using a competing talker paradigm, where different stories were presented to each ear at 0 dB, making it a more complicated listening task compared with the current study, where only a single story was presented. Overall, the current study suggests that cortical tracking of speech has good test–retest reliability, with differences in reliability based on methodological choices.

### Stimulus

One goal of cortical tracking research is to improve ecological validity of speech–language assessment. Although audiobooks are more naturalistic than typical constrained speech–language stimuli, podcasts offer an even higher level of naturalism, even incorporating elements like laughter and clapping. What makes podcasts more natural, however, also makes them noisier, potentially impacting test–retest reliability. Indeed, test–retest reliability was higher for the audiobook than the podcast. Factors to consider are that the audiobook was narrated by a professional speaker with clear articulation, while the podcast was not. Furthermore, the podcast had additional sounds such as audience laughter or clapping and other background noise. These differences in stimulus acoustics could have resulted in slightly lower cortical tracking and more variability across sessions. Additionally, the flow of information in the audiobook was dense, hence ensuring sustained attention in both sessions, while the information flow in the podcast was slower, potentially disengaging the listeners in the second session. While the more “controlled” audiobook stimulus had better test–retest reliability, it is also important to develop methods that can provide strong test–retest reliability to more naturalistic stimuli to ensure good ecological validity. Lastly, it is important to note that participants heard the same story and podcast in both sessions. This was an intentional choice to minimize the impact of stimulus characteristics on reliability across sessions. Although this could have negatively impacted attention to the stimulus in the second session, given the strong ICCs observed, it is unlikely that this occurred.

### Frequency band

Tracking in different EEG bands provides insight into neural mechanisms underlying the processing of various stimulus features (Etard and Reichenbach, 2019; Gnanateja et al., 2022). The delta band (1–4 Hz) has been reported to process aspects of speech that unfold relatively slowly, such as words and phrases, whereas the theta band (4–8 Hz) purportedly processes speech at the syllabic rate (Ding et al., 2015, 2017a,b; Gnanateja et al., 2022). Given the potential clinical utility of isolating specific processes of interest, we thus examined test–retest reliability separately within these frequency bands. We observed better reliability for cortical tracking in theta than delta or full bands. This is likely due to the fact that the most prominent energy in the speech envelope is concentrated in the theta range (Ding et al., 2017a,b; Poeppel and Assaneo, 2020), so cortical tracking of the envelope is also primarily concentrated in the theta range. This frequency region also corresponds to syllabic rhythm of speech, and neural oscillations in theta frequencies are thought to be important for encoding this syllabic information (Giraud and Poeppel, 2012; Doelling et al., 2014). We thus suggest that theta reliability is higher because it indexes a lower-level segmentation process. These segments then are joined together to form higher-level units of linguistic representation, which are probably processed more by the delta band, which responds more to lexical and semantic aspects, thus being more variable. Supporting this, Slaats et al. (2023) found that the influence of word frequency on neural responses varied depending on whether the word was presented in isolation versus in a sentence, with the effect primarily observed in the delta band. Furthermore, delta band is impacted more by higher-order cognitive factors (e.g., attention, effort). One additional explanation is that, because participants had heard the story previously, they were less attentive in the second session, or did not engage the same linguistic processing resources as in the first session. This would be more likely to impact the delta band, as previous research has reported that delta, but not theta, tracking of the envelope is sensitive to attentional manipulations. Specifically, delta band tracking of the speech envelope was reduced when participants were instructed to focus on a visual stimulus (a movie with subtitles) and ignore the auditory stimulus (Vanthornhout et al., 2019). Because theta rhythm is directly evident in the stimulus, there is no need for explicit hierarchical object formation driven by active attentive top–down mechanisms (Ding et al., 2015, 2017a,b). In our ongoing research on test–retest reliability in clinical populations, we are indirectly assessing attention to the story by including comprehension questions in both sessions, allowing for an examination of whether accuracy changes from Session 1 to Session 2. Lastly, delta is slower frequency and thus more susceptible to noise-related artifacts.

### Stimulus feature

Examining different stimulus features can help researchers understand the fine-grained differences in processing multidimensional speech features (Brodbeck et al., 2018; Broderick et al., 2018; Xie et al., 2023). Among the speech features assessed, the multiband envelope showed better reliability than the broadband envelope, and the envelope derivatives showed better reliability than the envelopes alone. The multiband envelope accounts for more variance in EEG data by better capturing band-specific differences in speech compared with the broadband envelope (Di Liberto and Lalor, 2017; Prinsloo and Lalor, 2022). Furthermore, synthetic speech prepared with a single broadband envelope shows poorer intelligibility than synthetic speech using multiband envelopes (Shannon et al., 1995; Smith et al., 2002), suggesting that listeners rely on multiband envelope cues to perceive speech. Thus, accurate and reliable modeling of neural responses requires features that are meaningful for speech perception. Moreover, the first derivatives of the broadband and multiband envelope signify stimulus onsets or edges which are vital for parsing speech into stimulus rhythm-driven segments (Oganian and Chang, 2019; Chalas, et al., 2022). This likely contributes to the better reliability observed for the derivatives. Overall, this study suggests that the most reliable stimulus feature for cortical tracking is the multiband envelope derivative.

### Reference electrode

Understanding how reference electrode choices influence reliability can help researchers make informed decisions that maximize test–retest reliability. The choice of reference electrode had a major impact on test–retest reliability, with the average mastoid reference showing better reliability than the CAR. The average mastoid reference is one of the most common reference strategies used for cortical tracking of speech acoustics (Crosse et al., 2016; Di Liberto et al., 2018; Decruy et al., 2019; Devaraju et al., 2021; McHaney et al., 2021), aligning well with the widely used vertical montage that emphasizes the vertically oriented equivalent current dipole generated at the auditory cortex (Näätänen and Picton, 1987). However, some studies have used CAR (Kalashnikova et al., 2018; Dial et al., 2021; Attaheri et al., 2022; Gillis et al., 2023) or reference-free approaches (Lalor and Foxe, 2010). Although the choice of reference electrode does not seem to influence cortical tracking prediction accuracy when averaged across scalp electrodes, the channel-specific prediction accuracies will vary (Brodbeck et al., 2023). Most studies on cortical tracking use a subset of recording electrodes to average the prediction accuracies to obtain a robust estimate of tracking that is not affected by noise in less important electrodes (Di Liberto et al., 2018; Decruy et al., 2019; Devaraju et al., 2021; McHaney et al., 2021). As such, the choice of reference electrode should be based on the electrodes that are being used for calculating the prediction accuracy. Considering the relatively better reliability observed in the current study for the average mastoid reference, we recommend researchers use the average mastoid reference for future work examining cortical tracking of the speech envelope.

### Conclusion and future directions

The findings of this study support the clinical utility of the method. The findings will allow researchers to more effectively design and implement studies on cortical tracking in neurotypical adults and adults with hearing and language disorders and provide a foundation for interpreting previous research. Characterizing the range of variability across sessions will also help with setting benchmarks for these measures to be used clinically for assessment or tracking treatment outcomes. That said, for populations with language disorders, this method will likely be of most value when estimating cortical tracking of linguistic features of speech, and future work should query test–retest reliability of linguistic feature tracking.

## Synthesis

Reviewing Editor: Niko Busch, Westfalische Wilhelms-Universitat Munster Decisions are customarily a result of the Reviewing Editor and the peer reviewers coming together and discussing their recommendations until a consensus is reached. When revisions are invited, a fact-based synthesis statement explaining their decision and outlining what is needed to prepare a revision will be listed below. The following reviewer(s) agreed to reveal their identity: Edmund Lalor. Note: If this manuscript was transferred from JNeurosci and a decision was made to accept the manuscript without peer review, a brief statement to this effect will instead be what is listed below.

# Synthesis

We have now received comments from two expert reviewers. Both reviewers provided generally positive evaluations, emphasizing the importance and relevance of testing the test-retest reliability of cortical speech tracking, as well as the quality of the experimental design. However, the reviewers provided several constructive suggestions for improving the manuscript. Many of these points concern requests for further explanations and for making the text and figures more accessible. I encourage you to address these comments carefully and comprehensively.

Additionally, I strongly recommend making your data and analysis code publicly available. Please include the link to the relevant repository in the Methods section of your manuscript under a dedicated section titled "Code Accessibility." For a clear example, please refer to:

Microsaccades Track Location-Based Object Rehearsal in Visual Working Memory

Eelke de Vries, Freek van Ede

eNeuro, 4 January 2024, 11 (1) ENEURO.0276-23.2023; DOI: 10.1523/ENEURO.0276-23.2023

# Reviewer 1

The manuscript provides important advances in the field by examining the reliability of cortical tracking of the speech envelope across various conditions, offering valuable insights into its robustness.

This study investigates the test-retest reliability of cortical tracking of the speech envelope, a non-invasive method for assessing speech processing in the brain. It examines various factors that could influence reliability, including different speech stimuli (audiobook vs. podcast), stimulus features (broadband and multiband envelopes), reference electrodes (average mastoid vs. common average reference), and EEG frequency bands (delta, theta, full). The results show moderate to good reliability for most parameters, indicating that cortical tracking of the speech envelope is a reliable tool for studying speech processing. These findings are valuable for researchers designing studies on cortical tracking in neurotypical adults and those with language disorders. However, I have a few considerations and critiques that should be addressed to refine the interpretation and application of the results.

1."Audio was presented through Sennheiser HD280 Pro headphones. The volume was adjusted as needed to the participants' comfort levels, typically between 65 - 75 dB SPL for the audiobook and 55 - 60 dB SPL for the podcast. All participants first listened to the audiobook, followed by a break for the participant's chosen duration before listening to the podcast."

How was the sound level verified during the experiment? What method was used to measure the sound levels? Were the stimuli presented in full scale in the digital domain before adjusting the loudness? The 10 dB difference in loudness levels between the audiobook and podcast is notable, as a difference of this magnitude is generally associated with a perceived doubling of loudness. In other words, participants likely perceived the audiobook as twice as loud as the podcast, which could influence the TRFs, prediction accuracy and reliability. While in the discussion, you suggest that the slightly different TRF/prediction accuracy could be attributed to more ecologically valid stimuli = more noise or the articulation of a professional speaker, this difference could also be due to the loudness discrepancy. Would it be possible to control for loudness in your statistical model? Perhaps extracting the perceived loudness or the RMS of the presented signal and including it as a regressor in the model would help account for this factor.

2. The figure and its caption should be more self-explanatory. Currently, the figure caption is missing some essential details. Could you please ensure that the figure caption and the figure itself clearly indicate the TRF for electrode Cz? Additionally, the time window for the topographic maps (topos) should be specified. What are the errorbars around ICC? This information is crucial for readers to fully understand the figure and its context.

3. The topographic maps generally look fine, but they may not be sufficiently auditory in nature. Have you checked the topographies for the TRF beta weights? Are they showing the typical central/focal topography for P1 and the N1 central-to-frontal topography expected for auditory processing?

4. "One additional explanation is that, because participants had heard the story previously, they were less attentive in the second session or did not engage the same linguistic processing resources as in the first session."

You can check this, right? You have behavioral data from the comprehension questions-why not use it to support or test this explanation? In general, I feel that the behavioral data is underutilized throughout the manuscript and could provide valuable insights into the participants' engagement and attention during both sessions.

# Reviewer 2

This manuscript has the potential to make a useful contribution to the literature on speech electrophysiology - the TRF approach has become relatively common, and understanding how reliable it is has implications for applied research. The manuscript also has the potential to add some value beyond other recent related papers (e.g., Panela et al). That all said, I think the manuscript would benefit from some revisions.

This manuscript presents research aimed at assessing the test-retest reliability of EEG responses to continuous, naturalistic speech. The authors collect EEG to a podcast and an audiobook from participants in different testing sessions separated by a few weeks. They then compare the temporal response functions and prediction accuracies based on those TRFs across sessions. They do this for TRFs fit using the broadband speech envelope, the broadband speech envelope derivative, the multiband speech envelope, and the multiband speech envelope derivative. They also do so after referencing the data to either the mastoids or a common average reference. And they also do it after filtering the EEG between 1-8 Hz or 1-4 Hz or 4-8Hz. In general, they report that the TRFs and prediction accuracies are relatively consistent across sessions.

This manuscript has the potential to make a useful contribution to the literature on speech electrophysiology - the TRF approach has become relatively common, and understanding how reliable it is has implications for applied research. The manuscript also has the potential to add some value beyond other recent related papers (e.g., Panela et al). That all said, I think the manuscript would benefit from some revisions.

Main comments:

1) One thing that I thought was - almost - entirely lacking from the manuscript was any discussion of how much data is required to obtain reliable TRFs in the first place. I say almost because the authors do touch on the issue indirectly with their SNR analysis. But some further discussion of this seems essential. If one were to collect a limited amount of data in each testing session, it is likely that the TRFs would not correspond very well at all. And if one were to collect hours of data in each testing session, it is entirely possible that the TRFs would be significantly higher across sessions. I know that the SNR analysis is supposed to speak to this - but it does not feel entirely satisfactory. The fact that it has no significant effect in the ANOVA is not strong proof that noise is not playing some kind of role in the ICC values. So I think some further discussion of this issue is warranted. The authors might even consider conducting an analysis of test-retest reliability as a function of how much data is used to fit the TRFs in each session. Presumably if you only used half or a quarter of your data in each session you would see the ICCs drop. And you could then plot the ICCs as a function of amount of data to get a sense of how near the asymptote you are with your analysis.

2) I have to say that I found the results section and figures impossible to understand - really very frustrating. Specifically, I thought the description of the ICC calculations was very sparse and confusing. I had no idea what is depicted in the TRF ICC column or the Prediction Accuracy ICC column in Figs 3-7. We are told in the text that ICC was calculated for each participant (within-subjects ICC) and separately for each electrode channel for each stimulus, stimulus feature, reference condition, and EEG frequency band. As such, I have no idea what the plots in the TRF ICC column are. They are within subject?! How are they time-resolved? What is the correlation over? Not electrodes because we are told that it is done separately for each electrode. The plots make no sense based on the text. Furthermore, the column on Prediction Accuracy ICC makes no sense. How do you end up with one plot if it within subject. Relatedly, I have no idea what a Bland-Altman plot is and there is no description in the caption or the text to explain what it is or what the axes even mean. What does "Average" mean on the x-axis? No idea.

3) Somewhat related to the previous point, I also failed to understand why participants are included as a random intercept in an analysis that is supposed to assess prediction accuracy between sessions. Surely the only way to assess consistency from one session to the next is to show that participants who have large prediction accuracies in session 1 also have large prediction accuracies in session 2. Does including participant as a random intercept not make this impossible? Again, I just don't know how you are assessing correlation/reliability at all. It is impossible to glean from the text.

4) Regarding the frequency based analysis - I must declare a bias - I am not at all convinced that EEG tracking of speech has anything to do with EEG oscillatory bands. The strength of tracking in these bands is going to be strongly influenced by the strength of the modulations in the stimulus at those frequencies. So - a bit like point number 1 above - it is somewhat difficult to appreciate the meaning of reliability numbers without understanding the properties of the stimulus one used to elicit those numbers. For example, if I were to take an extreme case of a stimulus that only had modulations between 1 and 4 Hz, then I would expect near zero test-retest reliability in the "theta band". This might be another thing that is worth discussing - it may also be worth showing the modulation spectra of your stimuli so readers can appreciate what the frequency analysis means.

Minor comments:

1) The way it is written it seems like participants listened to the same story and the same podcast on their two visits. Any specific reason for that? Responses the second time around may differ because it is a repetition and people may remember some of the content. I guess maybe not a huge deal because the sessions are separated by weeks, but it seemed a slightly odd choice to repeat stimuli. It's not like the repetition is part of the analysis per se. It could have been separate sections from the same audiobook. Might be worth a small comment (or not).

2) The age range seemed huge. Anything interesting in terms of reliability as a function of age?

3) I thought adding a brief sentence to explain the principle underlying ASR analysis might have been worth adding for those who are not familiar with it.

4) I wasn't quite sure what was meant by the line "The median prediction accuracy for every model was better than the chance prediction accuracy." This sounds like the statistics didn't actually work out - just that the median value of the prediction accuracies was higher than the (median?) of the chance distribution?? I don't know what the line means.

5) In the discussion paragraph on "Stimulus", you could consider adding some speculation or reference to other speech/language features that people have been using with TRFs. Or not.

Typos:

1) "its", not "it's" on line 218.
